# Significance of monitoring the levels of thyroid hormone antibodies and glucose and lipid metabolism antibodies in patients suffer from type 2 diabetes

**DOI:** 10.1515/med-2023-0876

**Published:** 2023-12-20

**Authors:** Xuefang Liu, Youyan Qiu, Dandan Chen, Jingni Xiong, Biwen Xia, Caiqin Chen, Suyan Li

**Affiliations:** Department of Endocrinology, The Fourth Affiliated Hospital of Guangzhou Medical University, Guangzhou 511300, Guangdong Province, PR China; Department of Endocrinology, The Fourth Affiliated Hospital of Guangzhou Medical University, No. 1, Guangming East Road, Zengjiang Street, Zengcheng District, Guangzhou 511300, Guangdong Province, PR China

**Keywords:** type 2 diabetes, thyroid hormone, antibodies to glucose and lipid metabolism, quality of life, monitoring value

## Abstract

The association of thyroid hormone antibodies and glycolipid metabolism indicators with Type 2 diabetes mellitus (T2DM) was explored. As the disease worsens, the levels of thyroglobulin antibody (TGAb), thyroid peroxidase antibody (TPOAb), and thyroid-stimulating hormone (TSH) was increased, and the levels of total tri-iodothyronine (TT3) and total thyroxine (TT4) was decreased (*P* < 0.001). The severe, medium, and light group had higher level of high-density lipoprotein (HDL), lower level of total cholesterol (TC), low-density lipoprotein (LDL), glycosylated hemoglobin (HbA1c), triacylglycerol (TAG), and fasting blood sugar (FBG) than the control group (*P* ＜ 0.05). The level of HDL was lower in the severe group than the light group and the medium group, but the levels of TC, LDL, HbA1c, TAG, and FBG were increased with the progress of T2DM (*P* ＜ 0.001). The levels of TGAb, TPOAb, and TSH in patients with T2DM were positively correlated with the levels of TC, LDL, HbA1c, TAG, and FBG (*P* < 0.05), and were negatively correlated with HDL levels (*P* < 0.05). The life quality score was lower in the severe group than the light and the medium group (*P* < 0.001). Among the above indicators, the predictive value of TT3, TT4, and HbA1c in T2DM was better. Clinically, detecting the levels of thyroid hormone antibodies and glycolipid metabolism indicators had a certain predictive value for the severity of T2DM.

**Main findings:** The results of this study found that the thyroid hormone antibody and glycolipid metabolism levels in T2DM patients were abnormal, and had different degrees of impact on the quality of life of patients. Thus, monitoring these indicators had certain predictive value for the severity of the disease, and also had a certain degree of suggestive effect on the evaluation of diabetic vascular complications. Clinically, attention should be paid to the screening of thyroid disease in diabetic patients, and the assessment and prognosis of thyroid function on diabetes, the control of diabetes, and the prevention and treatment of complications have important clinical significance.

## 1 Introduction

Type 2 diabetes mellitus (T2DM) is an insulin resistance disease, which is a metabolic disorder caused by insufficient insulin secretion in the body. Patients will have abnormal thyroid hormone and insulin secretion [1]. Thyroid hormone can be used as an important regulator of energy consumption and systemic metabolism. The disordered hormone will affect the gland function, and even affect the function of multiple organs in the body in severe cases. The uncontrolled insulin regulation of T2DM patients leads to disorder of protein, sugar, and fat metabolism, which in turn leads to thyroid dysfunction, which seriously affects glucose and lipid metabolism, increases the incidence of complications, and threatens the life safety of patients [2].

In recent years, clinical studies have found that thyroid antibodies are elevated in patients with T2DM. Thyroid antibodies mainly include thyroid peroxidase antibody (TPOAb) and thyroglobulin antibody (TGAb), of which TGAb is the most significant. Excessive antibody attacks on one’s own tissues leads to damage to thyroid follicles, which in turn causes changes in the normal rhythm of hormone secretion, eventually leading to thyroid dysfunction [3]. If the thyroid axis regulation, hypothalamus, and adenohypophysial function of the patient are abnormal, thyroid hormone level will be affected. This phenomenon will alter with the change of the patient’s hormone level, and the thyroid hormone level will continue to increase with the progress of the disease. In addition, studies have confirmed that the levels of free thyroxine (FT4) are higher in patients with type 2 diabetes and hypertension than in non-diabetic populations [4]. Clinically, thyroid dysfunction can cause blood pressure to rise. Thyroid hormone, as an important regulator of energy metabolism, can improve the excitability of various systems, increase cardiac excretion, affect sodium ions, as well as regulate the fluid regulation system for the balance of water and electrolytes. Increased myocardial contractility, heart rate and circulating blood volume due to increased thyroid hormone can lead to elevated blood pressure. Hyperlipidemia caused by T2DM is one of the main risk factors for diabetic macroangiopathy, and metabolic syndrome components such as T2DM dyslipidemia, hyperglycemia, and insulin resistance can accelerate the development of atherosclerosis [5]. Therefore, some diabetic patients still develop diabetic macrovascular lesions even if they have ideal blood sugar control.

This study aimed to analyze the changes and correlation of thyroid hormone antibody and glycolipid metabolism level in T2DM patients, explore the pathogenesis of T2DM metabolism disorders, and provide a theoretical basis for finding new treatment pathways.

## 2 Materials and methods

### 2.1 General information

A total of 152 patients with T2DM admitted to our hospital from January 2019 to December 2021 were selected as the research group, and the patients were divided into three groups, including severe group (*n* = 26), mild group (*n* = 74), and the medium group (*n* = 52). A total of 152 healthy subjects were taken as controls at the same time. Inclusion criteria: (1) those who conform to the diagnostic criteria in the “T2DM Diagnostic Guidelines” [6], (2) those who have complete information and signed the informed consent form, and (3) those who have normal cognitive function and high compliance. Exclusion criteria: (1) those who received drug therapy in the first 6 months of the study, (2) those with co-infection and malignant tumors, (3) those with abnormal liver and kidney function, (4) those with immune system diseases, and (5) those with communication disorders or mental illness. There was no difference in general data between the two groups (*P* > 0.05). The study was approved by the hospital ethics committee.

### 2.2 Detection/evaluation methods for each indicator

One day before the test, all patients were fasted. Then, 3 mL of fasting cubital median venous blood was drawn in the morning of the day after admission, and centrifuged at 3,000 rpm to obtain the upper serum.

(1) Indicators of glycolipid metabolism: The Hitachi automatic biochemistry analyzer was used to detect the level of high-density lipoprotein (HDL), total cholesterol (TC), low-density lipoprotein (LDL), glycosylated hemoglobin (HbA1c), triacylglycerol (TAG), fasting blood sugar (FBG), and other glycolipid metabolism indicators of all the study subjects. TC and TG levels were determined by enzyme reagent method, and LDL-C and HDL-C were determined by direct method on the automatic biochemical analyzer of Beckman Company, United States, using reagents matching the instrument to measure, with the intra-batch and inter-batch CV <1%. The experiments were operated by a full-time laboratory personnel. Measurement of HbA1c: The measurement was conducted using the British Drew DS360 automatic glycated hemoglobin automatic analyzer and supporting reagents determination, and cation exchange high performance liquid chromatography method determination, with the intra-batch and inter-batch CV <1%. Experimental steps: (1) A certain amount of whole blood sample were injected into the sampling device by the sampling needle. (2) Hemoglobin (Hb) in red blood cells was released and diluted by diluent, and then the diluted hemolyzed sample was injected into the ion exchange column by a high-pressure pump. (3) After the sample was added from the tip of the exchange column through a filter, it was eluted by three different concentrations of salt elution buffer, moving the sample downward. (4) The detector of the instrument detected the absorbance values of the separated HbA1c, HbF, and HbA1 components, compared them with the absorbance values of the HbA1c standard, and analyzed and calculated the results.
(2) Thyroid hormones: The electrochemiluminescence automatic immunoassay analyzer (Roche Elecsys 2010) was used to detect the level of TGAb, total tri-iodothyronine (TT3), anti-TPOAb, total thyroxine (TT4), and thyroid-stimulating hormone (TSH) of all the study subjects. The detection was conducted by solid phase chemiluminescence immunoassay, with the intra-batch and inter-batch CV <1%. Basic principle: The ACCESS system used magnetic particles as solid-phase carriers, based on immunoassay methods such as alkaline phosphatase as luminescent competition, sandwich, and antibody detection. Experimental steps: (1) Discharge: the sample number was into the sample tray. (2) Put the reagents used in the reagent tray according to the test items and observe. (3) Freely arrange the program, press the “START” button according to the compiled worksheet, automatically place the reaction cup in the controller of the microcomputer, automatically add samples and reagents, and the incubation time is generally 2.5–7.5 min. Results were determined from start up to 16 min. The analyzer automatically calculated the reported results. Then, the results were reported every 20 s, and three results were reported every minute.
(3) Quality of life: The SF-36 scale was used to evaluate mental health, physical function, energy, general health, physical pain, social function, emotional function, and physiological function. The scale included eight dimensions with a total score of 100 points. Scores were proportional to life.

### 2.3 Statistical processing

The test results were entered into SPSS 20.0 software. The Kolmogorov–Smirnov test method was used to test whether the variables of the measurement data conformed to the normal distribution, and the measurement data conforming to the normal distribution were expressed in the form of (*x̄* ± *s*). The *t*-test was used for measurement data that conformed to the normal distribution, and the rank sum test (Wilcoxon’s test) was used for measurement data that did not conform to the normal distribution. One-way ANOVA was used for the multi-group comparison. The LSD-*t* test was used for the difference between multiple groups, and Tamhanes’s T2 test was used for the variance uniformity. Enumeration data were expressed in the form of (%), and analyzed by *χ* ^2^ test. The correlation between thyroid hormone antibodies and glycolipid metabolism levels was determined by the Pearson analysis. The clinical value of thyroid hormone indexes and glucose metabolism indexes in predicting the occurrence of diabetes was analyzed by receiver operating characteristic (ROC) curve. *P* < 0.05 indicated statistical significance.

## 3 Results

### 3.1 Analysis of general data of the patients

**Figure 1:**
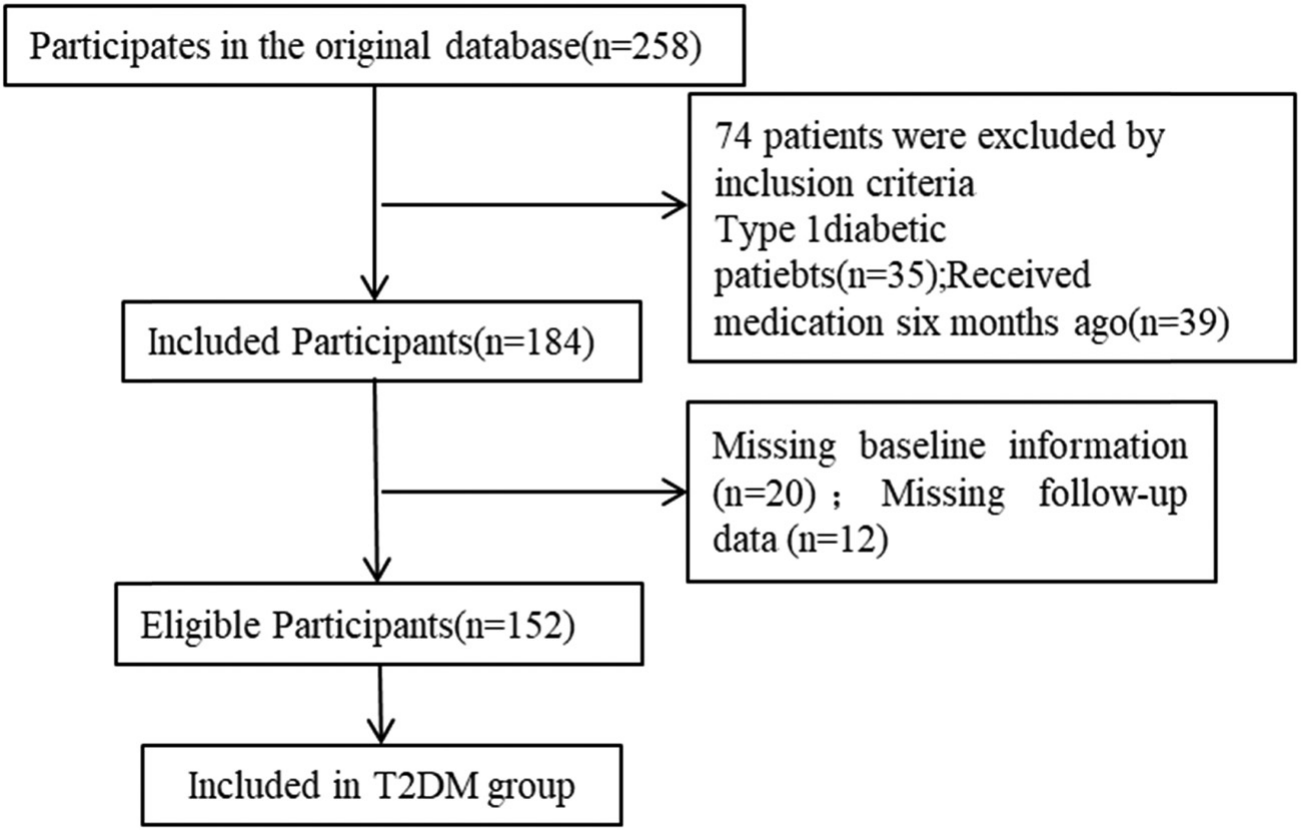
Flow chart of general data selection.

The selection flow chart of 152 patients with T2DM is shown in Figure 1. There were no significant differences in general data including sex, age, and BMI between the study group and the control group (*P* > 0.05, Table 1).

**Table 1:** Comparison of general data (*x̄* ± *s*), [*n* (%)]

Normal information		The control group (*n* = 152)	The study group (*n* = 152)	*χ* ^2^/*t*	*P*
Gender	Male	93 (61.2)	92 (60.5)	0.635	0.524
	Female	59 (38.8)	60 (39.5)		
Age (year)		58.64 ± 5.12	58.81 ± 5.26	0.752	0.835
BMI (kg/m^2^)		27.32 ± 4.28	27.29 ± 4.25	0.424	0.387
Blood pressure	Systolic pressure	128.36 ± 9.54	128.38 ± 9.55	0.759	0.415
	Diastolic pressure	85.24 ± 6.17	85.21 ± 6.19	0.042	0.966

### 3.2 Comparison of autoantibodies and thyroid hormone levels between the two groups

The levels of TT3 and TT4 in the study group were lower than those in the control group, and the levels of TGAb, TPOAb, and TSH were higher than those in the control group with prominent differences (*P* < 0.001, Table 2).

**Table 2:** Comparison of autoantibodies and thyroid hormone levels (*x̄* ± *s*)

Groups	TGAb (U/mL)	TT3 (nmol/L)	TPOAb (U/mL)	TT4 (nmol/L)	TSH (μIU/L)
The control group (*n* = 152)	22.45 ± 2.16	2.84 ± 0.28	12.44 ± 1.18	83.02 ± 8.39	1.94 ± 0.17
The study group (*n* = 152)	31.79 ± 3.12	1.25 ± 0.14	16.85 ± 1.63	80.84 ± 8.36	2.97 ± 0.28
*t*	21.541	32.658	18.414	21.354	24.156
*P*	<0.001	<0.001	<0.001	<0.001	<0.001

### 3.3 Comparison of autoantibodies and thyroid hormone levels in patients with different severity of disease

The levels of TGAb, TPOAb, and TSH in severe, medium, and light groups were higher, while those of TT3 and TT4 in severe, medium, and light group were lower than those in the control group (*P* ＜ 0.05). In comparison with the light group and the medium group, the severe group had higher levels of TGAb, TPOAb, and TSH with lower levels of TT3 and TT4. The difference was distinct (*P* < 0.001, Table 3).

**Table 3:** Comparison of autoantibodies and thyroid hormone levels in patients with different severity of disease (*x̄* ± *s*)

Groups	TGAb (U/mL)	TT3 (nmol/L)	TPOAb (U/mL)	TT4 (nmol/L)	TSH (μIU/L)
Severe group (*n* = 26)	33.38 ± 3.27	1.11 ± 0.13	18.96 ± 1.82	81.80 ± 8.23	2.61 ± 0.24
Moderate group (*n* = 52)	30.51 ± 3.25	1.86 ± 0.18	16.71 ± 1.63	82.25 ± 8.38	2.12 ± 0.19
Light group (*n* = 74)	24.75 ± 2.44	2.63 ± 0.27	13.59 ± 1.29	82.92 ± 8.34	1.55 ± 0.14
Control group (*n* = 152)	22.45 ± 2.16	2.84 ± 0.28	12.44 ± 1.18	83.02 ± 8.39	1.94 ± 0.17
*F*	224.45	468.62	255.23	3.89	271.23
*P*	<0.001	<0.001	<0.001	<0.001	<0.001

### 3.4 Comparison of glucose metabolism indicators in two groups

The level of HDL in the study group was lower than that in the control group, while the levels of TC, LDL, HbA1c, TAG, and FBG were higher than those in the control group with outstanding differences (*P* < 0.05, Table 4).

**Table 4:** Comparison of glucose metabolism indicators in two groups (*x̄* ± *s*)

Groups	HDL (mmol/L)	TC (mmol/L)	LDL (mmol/L)	HbA1c (%)	TAG (mmol/L)	FBG (mmol/L)
The control group (*n* = 152)	1.45 ± 0.12	4.24 ± 0.42	2.66 ± 0.25	5.07 ± 0.48	1.28 ± 0.11	5.11 ± 0.49
The study group (*n* = 152)	1.02 ± 0.08	5.87 ± 0.56	3.29 ± 0.31	8.95 ± 0.88	2.31 ± 0.21	8.78 ± 0.86
*t*	24.812	19.863	13.325	17.228	20.754	16.581
*P*	<0.001	<0.001	<0.001	<0.001	<0.001	<0.001

### 3.5 Comparison of glucose and lipid metabolism levels in patients with different severity of disease

In step with the light group and the medium group, the severe group had lower levels of HDL and higher levels of TC, LDL, HbA1c, TAG, and FBG, and the difference was statistically significant (*P* < 0.001, Table 5).

**Table 5:** Comparison of glucose and lipid metabolism levels in patients with different severity of disease (*x̄* ± *s*)

Groups	HDL (mmol/L)	TC (mmol/L)	LDL (mmol/L)	HbA1c (%)	TAG (mmol/L)	FBG (mmol/L)
Severe group (*n* = 26)	0.96 ± 0.08	6.05 ± 0.58	3.51 ± 0.33	7.11 ± 0.89	2.53 ± 0.24	13.01 ± 1.27
Moderate group (*n* = 52)	1.03 ± 0.08	5.61 ± 0.54	3.22 ± 0.31	6.52 ± 0.76	2.21 ± 0.19	10.64 ± 1.03
Light group (*n* = 74)	1.34 ± 0.12	1.61 ± 0.14	2.77 ± 0.28	5.57 ± 0.53	1.61 ± 0.14	7.02 ± 0.68
Control group (*n* = 152)	1.45 ± 0.12	4.24 ± 0.42	2.66 ± 0.25	5.07 ± 0.48	1.28 ± 0.11	5.11 ± 0.49
*F*	280.23	1317.47	107.56	140.11	878.00	1316.26
*P*	<0.001	<0.001	<0.001	<0.001	<0.001	<0.001

### 3.6 Correlation between the thyroid hormone levels and glycolipid metabolism in patients with T2DM

The level of HDL was higher in severe, medium, and light groups, while those of TC, LDL, HbA1c, TAG, and FBG in severe, medium, and light groups were lower than those in the control group (*P* ＜ 0.05). The levels of TGAb, TPOAb, and TSH in patients with T2DM were positively correlated with the levels of TC, LDL, HbA1c, TAG, and FBG (*P* < 0.05), and were negatively correlated with HDL levels (*P* < 0.05, Table 6). The levels of TT3 and TT4 in patients with T2DM were negatively correlated with the TC, LDL, HbA1c, TAG, and FBG levels (*P* < 0.05), and positively correlated with the level of HDL (*P* < 0.05, Table 6).

**Table 6:** Correlation between the thyroid hormone levels and glycolipid metabolism in patients with T2DM

Indicators	TGAb		TT3		TPOAb		TT4		TSH	
	*r*	*P*	*r*	*P*	*r*	*P*	*r*	*P*	*r*	*P*
HDL	−0.341	0.002	0.390	0.001	−0.360	0.001	0.319	0.004	−0.396	0.001
TC	0.228	0.042	−0.285	0.011	0.276	0.013	−0.577	0.001	0.312	0.005
LDL	0.269	0.016	−0.313	0.005	0.241	0.031	−0.351	0.001	0.353	0.001
HbA1	0.226	0.044	−0.402	0.001	0.223	0.047	−0.312	0.005	0.286	0.010
TAG	0.227	0.043	−0.279	0.012	0.366	0.001	−0.449	0.001	0.356	0.003
FBG	0.251	0.025	−0.374	0.001	0.381	0.001	−0.392	0.001	0.353	0.001

### 3.7 Comparison of quality of life between the two groups

The score of quality of life in the study group was lower than that of the control group, and the difference was distinct (*P* < 0.001, Table 7).

**Table 7:** Comparison of quality of life (score, *x̄* ± *s*)

Groups	Mental health	Physiological function	Energy	General health	Body pain	Social function	Emotional function	Physiological function
The control group (*n* = 152)	86.34 ± 12.11	84.24 ± 11.32	80.58 ± 11.63	82.75 ± 12.93	80.71 ± 11.62	81.76 ± 10.87	83.62 ± 9.43	78.51 ± 4.68
The study group (*n* = 152)	72.32 ± 10.53	70.61 ± 9.62	71.56 ± 8.41	71.36 ± 7.62	70.85 ± 6.43	73.61 ± 5.13	72.56 ± 5.17	68.92 ± 4.83
*T*	25.314	23.421	24.782	16.024	22.571	23.654	21.647	20.414
*P*	<0.001	<0.001	<0.001	<0.001	<0.001	<0.001	<0.001	<0.001

### 3.8 Comparison of quality of life among patients with different severity of illness

Compared with the light group and the medium group, the severe group had a lower quality of life score, and the difference was arresting (*P* < 0.001, Table 8).

**Table 8:** Comparison of quality of life among patients with different severity of illness (score, *x̄* ± *s*)

Groups	Mental health	Physiological function	Energy	General health	Body pain	Social function	Emotional function	Physiological function
Severe group (*n* = 26)	71.51 ± 9.84	69.52 ± 9.25	71.11 ± 8.36	70.97 ± 7.42	70.26 ± 6.21	73.16 ± 4.88	72.65 ± 4.52	71.64 ± 4.39
Moderate group (*n* = 52)	75.34 ± 9.68	72.52 ± 9.57	73.14 ± 8.63	75.65 ± 7.28	76.13 ± 7.54	80.91 ± 6.36	75.24 ± 6.58	76.18 ± 6.64
Light group (*n* = 74)	84.13 ± 11.65	83.54 ± 10.82	79.67 ± 10.93	82.35 ± 12.51	80.18 ± 11.23	81.24 ± 10.56	82.44 ± 10.16	81.96 ± 10.37
*F*	24.328	25.741	23.925	19.582	24.524	22.785	23.541	21.756
*P*	<0.001	<0.001	<0.001	<0.001	<0.001	<0.001	<0.001	<0.001

### 3.9 ROC curve analysis of the clinical value of thyroid hormone indicators in predicting the occurrence of diabetes

**Figure 2:**
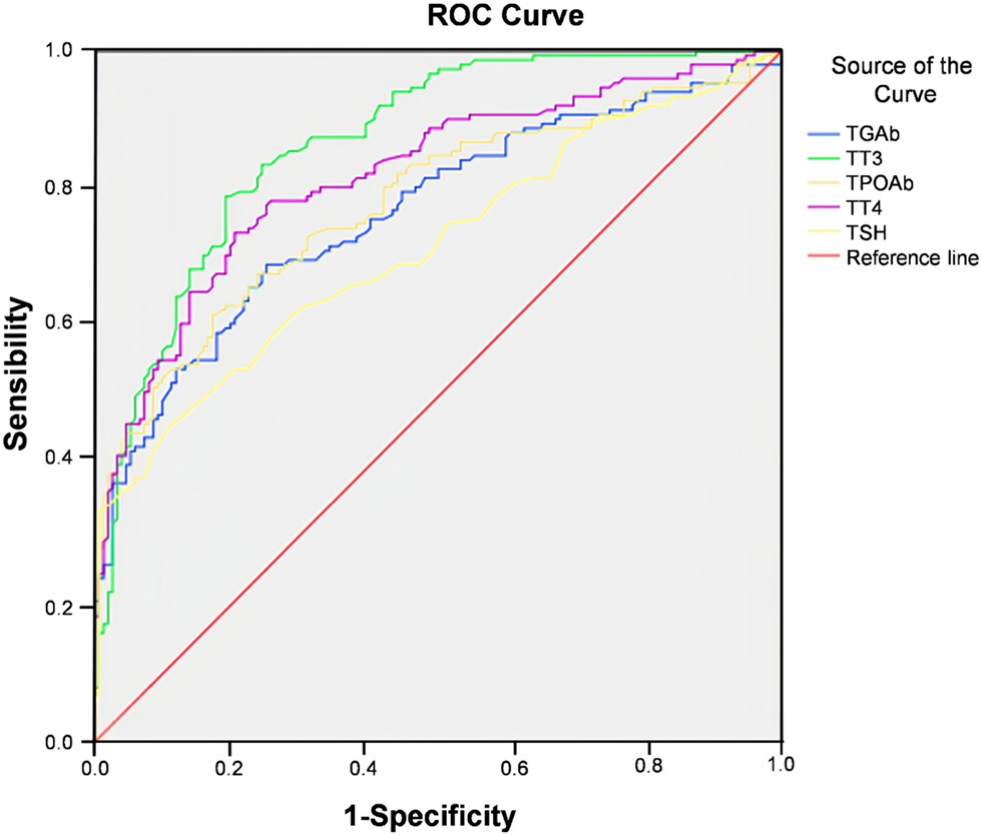
ROC curve of thyroid hormone indicators predicting the occurrence of diabetes.

The area under curve (AUC) of TGAb, TT3, TPOAb, TT4, and TSH for predicting diabetes were 0.766, 0.867, 0.779, 0.819, and 0.716, respectively. The predictive value of TT3 and TT4 was better than other indicators (Table 9, Figure 2).

**Table 9:** Clinical value of thyroid hormone indicators in predicting the occurrence of diabetes

Indicators	AUC	*P*	95% CI	Sensitivity (%)	Specificity (%)
TGAb	0.766	<0.001	0.713–0.820	91.40	77.60
TT3	0.867	<0.001	0.827–0.907	94.10	86.80
TPOAb	0.779	<0.001	0.726–0.931	88.80	72.40
TT4	0.819	<0.001	0.772–0.867	96.10	80.90
TSH	0.716	<0.001	0.658–0.773	78.90	58.60

### 3.10 ROC curve analysis of the clinical value of glucose and lipid metabolism indicators in predicting the occurrence of diabetes

**Figure 3:**
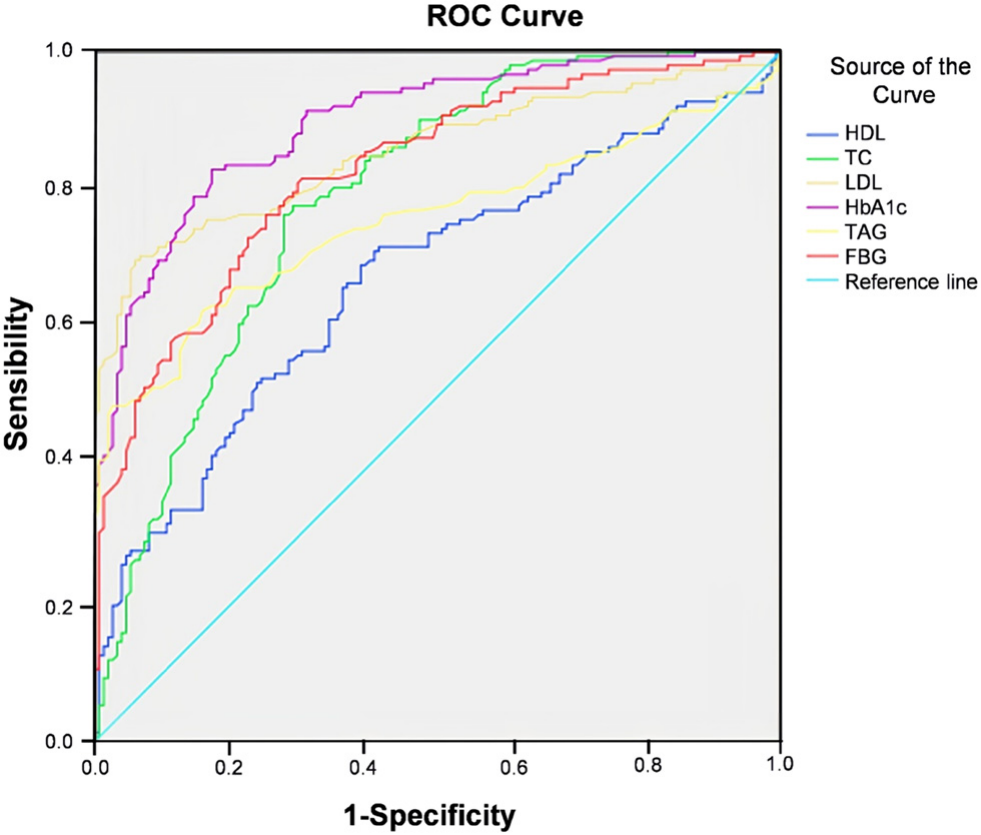
ROC curve of glucose metabolism indicators predicting the occurrence of diabetes.

The AUC values of HDL, TC, LDL, HbA1c, TAG, and FBG for predicting diabetes were 0.672, 0.791, 0.856, 0.898, 0.755, and 0.827, respectively. The predictive value of HbA1c was better than other indicators (Table 10, Figure 3).

**Table 10:** Clinical value of glucose metabolism indexes in predicting the occurrence of diabetes

Indicators	AUC	*P*	95%CI	Sensitivity (%)	Specificity (%)
HDL	0.672	<0.001	0.611–0.732	83.60	69.70
TC	0.791	<0.001	0.741–0.842	91.40	53.90
LDL	0.856	<0.001	0.812–0.899	96.70	85.50
HbA1c	0.898	<0.001	0.864–0.932	98.70	73.70
TAG	0.755	<0.001	0.699–0.812	87.50	78.90
FBG	0.827	<0.001	0.782–0.873	94.70	65.80

## 4 Discussion

The living habits of humans have undergone great changes with promoted living standards. The amount of sugar intake has increased significantly, resulting in an elevated incidence of T2DM. T2DM has a high incidence and is a clinical multiple endocrine disease with irreversible and chronic development characteristics. If patients maintain high blood sugar levels for a long time, it will affect glucose and lipid metabolism and cause various complications [7]. The study has found that T2DM is an important metabolic disease that causes microvascular and macrovascular complications, and that good control of T2DM is associated with reduced diabetes-related morbidity and mortality [8]. Diabetes and thyroid disease are two of the most common diseases in endocrinology and are closely related. Domestic and foreign studies have shown that patients with T1DM and T2DM may have thyroid dysfunction [9]. In addition to promoting growth and development and tissue differentiation, thyroid hormones can also affect the metabolism of sugars, fats, and proteins, and participate in the occurrence and development of T2DM. The previous study has shown that thyroid hormone levels can assess blood glucose and lipid metabolism in patients with T2DM [10]. Besides, the previous study has found that the T3, T4, FT3, and F4 levels in the T1DM group are lower than those in the T2DM group, and the rT3 level is higher than that in the T2DM group, which shows that the thyroid hormone levels of different types of diabetes are also different [11]. The related reasons may be related to the earlier onset of type 1 diabetes, the difficulty of controlling insulin levels, and the heavier clinical manifestations. The previous study has explored the relationship between thyroid hormone levels and blood glucose and lipid metabolism in children with normal thyroid function and new-onset T1DM, and the results show that TSH is positively correlated with LDL-C, TC, and TG, and negatively correlated with FBG and HbAlc, while FT3 is negatively correlated with TG, FBG, and HbAlc [12]. In addition, the study has shown that the incidence of thyroid dysfunction in adult T1DM patients is 21.30%, which is higher than that of the general population and T2DM patients, and also higher than that of children with T1DM [13]. The related reasons may be due to similar genetic defects and immune destruction between T1DM and autoimmune thyroid disease, or it may be age-related, with studies showing that the incidence of thyroid dysfunction increases with age [14]. Based on the principle of early detection and early treatment, appropriate diagnosis methods should be taken to predict the severity of the disease and select a targeted treatment. This is an important measure to ameliorate the living quality and ensure patient safety.

Patients with T2DM have obvious metabolic disorders, and the secretion of thyroid hormone antibodies in patients changes greatly, which will affect the ability of TSH transport. The normal secretion function of patients can also be inhibited, which is the main reason for the reduction of TT3 levels [15]. The reducing of metabolic function will greatly weaken the insulin function, reduce the activity of thyroxine deiodinase, interfere with the TSH transport function, and decrease the amount of T4–T3 conversion. The TT3 level will also be reduced with the aggravation of clinical symptoms [16]. This study analyzed the changes of autoantibodies and thyroid hormone levels in T2DM and healthy people in detail. The results displayed that the contents of TT3 and TT4 in the research group were fewer than the control, but the contents of TGAb, TPOAb, and TSH were larger than the control. The results are consistent with those of other scholars, which indicate that T2DM patients have reduced self-resistance and remarkable changes in thyroid hormone levels, which are quite different from those of healthy people. In addition, this study found that T2DM patients were accompanied by obvious abnormal thyroid hormone levels, and increased levels of thyroid antibodies. The more severe the disease, the greater the increase in antibody levels. Under the mediation of the reaction, the secretion of insulin decreases, which will affect the thyroid function, thereby increasing the content of antibody, aggravating the degree of systemic disease, which is not conducive to the normal life [17,18]. The previous study has shown that the higher level of HbA1c indicates the higher TGAb level in diabetic patients [19]. HbA1c is a reflection of blood glucose levels in the past 3 months and is one of the criteria for measuring blood sugar control in people with diabetes. Thus, the worse the blood sugar control the higher the positive rate of thyroid autoantibodies, which is also considered as the more severe the glucose metabolism disorder the greater the impact on the thyroid gland, eventually leading to elevated thyroid antibodies. Diabetes mellitus combined with thyroid autoimmune abnormalities induces disorders in blood glucose. Poor glycemic control in turn accelerates the progression of thyroid disease and increases thyroid antibodies due to the elevated levels of HbA1c [20]. Roa Dueñas et al. [21] have found that patients with hypothyroidism and low FT4 levels in the reference range have an increased risk of developing T2DM. From the above studies, it could be seen that serum thyroid hormone regulated insulin sensitivity by increasing the absorption of sugar and water compounds in the intestine, and blood sugar disorders also aggravated serum thyroid hormone abnormalities. In view of the close relationship between thyroid hormone levels and metabolic syndrome in patients with type 2 diabetes, early screening of thyroid function in elderly patients with T2DM is of great significance to delay the occurrence of complications of T2DM combined with metabolic syndrome.

Thyroid hormone antibodies secreted by the thyroid gland are of great significance to the metabolism of the body, which is beneficial to the growth and development of the body and affects the function of the nervous system [18]. The results of this study showed that the level of HDL in the study group was lower than that in the control group, and the levels of TC, LDL, HbA1c, TAG, and FBG were higher than those in the control group. The results confirmed that there was a big difference in the levels of glucose and lipid metabolism indicators between healthy people and T2DM patients, mainly because T2DM patients suffer from insulin resistance, hyperglycemia, and hyperinsulinemia. Patients are accompanied by obvious stress reactions, which exerts a strong influence on islet function. Besides, patients are prone to lipid peroxidation and the probability of diabetic macrovascular complications will increase memorably, which will have a greater impact on the patient’s prognosis. The decrease of HDL level and the increase of LDL and TC levels in T2DM patients can change the metabolic properties, and the large particle LDL has a higher chance of inducing atherosclerotic disease, which is the main reason for the changes in the level of glucose and lipid metabolism indicators [22]. Based on this research, this study conducted a more in-depth exploration and analyzed the changes of indicators to glucose and lipid metabolism in patients with different severity. The results showed that compared with the light group and the medium group, the heavy group had lower levels of HDL and higher levels of TC, LDL, HbA1c, TAG, and FBG. The results confirmed that with the aggravation of the disease, the levels of TC, LDL, HbA1c, TAG, FBG, and other glucolipid metabolism indicators exhibit an upward trend, and the HDL level displays a downward trend, which is closely related to the severity of the disease. With the aggravation of the disease, abnormal glucolipid metabolism phenomenon is more obvious. This trend of change is beneficial for the prediction of disease severity, and has important reference value for the formulation and adjustment of clinical treatment plans. In addition, the results of this study also showed that there was a correlation between thyroid hormone levels and glycolipid metabolism indicators in patients with T2DM. It has been found that serum TSH levels in the normal range were significantly correlated with TG, and for each increase in TSH l µIU/L”, the corresponding TG increased by 0.115 mmol/L [23]. Besides, the study has confirmed that in diabetic patients with poor glycemic control, as diabetes improves, blood glucose levels decrease, and FT3 levels gradually increase, further indicating that FT3 reduction is associated with poor glycemic control and diabetes severity [24]. Changes in thyroid hormone levels are closely related to the condition of T2DM, and when thyroid hormone levels are abnormal, they have different degrees of impact on the metabolism of sugar, protein, fat, water, electrolytes, etc., thereby accelerating the occurrence and development of T2DM and its complications [25]. Clinically, it is necessary to pay attention to the changes of glycolipid metabolism and thyroid hormones in patients with T2DM at any time, and be able to achieve regular testing, which is convenient for accurate assessment of the condition and the formulation of treatment plans, hoping to improve the efficiency of treatment, patients’ negative emotions, and the quality of life.

The study [26] has confirmed that there is a close relationship between glucose metabolism disorders and abnormal thyroid hormone levels. Hormone level disorders can lead to aggravation of the disease and induce a variety of dangerous events, such as cardiovascular disease, which affects the prognosis of patients and affects the living quality. This study found that the score of life quality in the study group was lower than the control and the score of life quality in the heavy group was worse. The results confirmed that the severity of the patient’s disease was positively related to the quality of life. With the increase in the severity of disease, the patient’s quality of life gradually decreased, affecting the patient’s normal work. Therefore, it is necessary to focus on the life quality of patients, and develop good intervention measures. In patients with T2DM, the incidence of metabolic syndrome is significantly higher than that of healthy people, and thyroid hormone is an important hormone that affects the metabolism of human material energy, making the relationship between the three very complex and closely related. Thus, T2DM should not only pay attention to blood sugar, but also pay attention to thyroid function and blood pressure, blood lipids, whether overweight and other metabolic syndrome related indicators, thereby putting forward screening and treatment strategies in line with the characteristics of our population, making individualized treatment plans after comprehensive assessment, improving the quality of life of patients, and delaying the occurrence of complications.

In conclusion, the levels of thyroid hormone antibodies and glucose and lipid metabolism in T2DM patients were different from the healthy controls. Clinical detection of thyroid hormone antibodies and glucose and lipid metabolism levels in T2DM patients has a certain predictive value for the severity of the disease, which can provide a theoretical basis for the formulation of clinical treatment measures, and is conducive to improving the quality of life of patients. However, this study only included T2DM patients admitted to our hospital, which cannot reflect the overall thyroid hormone antibody and glucose and lipid metabolism levels of domestic T2DM patients. In the follow-up studies, we will increase the experimental sample size, expand the experimental area, to further confirm the effect of thyroid hormone antibodies and glucose and lipid metabolism levels in the progression of T2DM.
